# Novel distance-progesterone-combined selection approach improves human sperm quality

**DOI:** 10.1186/s12967-018-1575-7

**Published:** 2018-07-20

**Authors:** Kun Li, Rui Li, Ya Ni, Peibei Sun, Ye Liu, Dan Zhang, Hefeng Huang

**Affiliations:** 1 Department of Reproductive Endocrinology, Women’s Hospital, School of Medicine, Zhejiang University, Hangzhou, 310006 China; 2 Department of Reproductive Physiology, Zhejiang Academy of Medical Sciences, Hangzhou, 310013 China; 3 Key Laboratory of Reproductive Genetics (Ministry of Education), Zhejiang University, Hangzhou, 310006 China; 4 International Peace Maternity and Child Health Hospital, Shanghai Jiao Tong University School of Medicine, Shanghai, 200030 China; 5 Institute of Embryo-Fetal Original Adult Disease, Shanghai Jiao Tong University School of Medicine, Shanghai, 200030 China

**Keywords:** Sperm, Sperm selection, Sperm motility, Sperm quality, Chemotaxis, Progesterone, Morphology, DNA fragmentation, Apoptosis, ART

## Abstract

**Background:**

Sperm selection is essential for the health of offspring conceived via assistive reproductive technology (ART). Various methods of sperm preparation for in vitro fertilization and intracytoplasmic sperm injection have been developed to acquire sperm with better quality and to avoid potential genetic disorders. However, current sperm processing and selection techniques bypass the natural selection that occurs during fertilization in vivo. The aim of this study was to present a novel distance-progesterone-combined selection approach with an original device based on the human female reproductive tract, and to report on its effectiveness based on sperm progressive motility, as well as chemotaxis.

**Methods:**

A novel device with long distance channels which mimicked the female human reproductive system was designed and fabricated. This ready-to-be-used device was developed using a progesterone gradient and human tube fluid media. Sperm swam for 150 min in the device under conditions of 37 °C air temperature with 5% CO_2_ after separation from seminal plasma via discontinuous Percoll gradient treatment. The selected sperm were assessed for normal morphology using Diff-Quik staining. A chromatin diffusion assay assessed sperm for DNA fragments and apoptosis was assessed using annexin V-fluorescein isothiocyanate/propidium iodide fluorescent staining.

**Results:**

Our distance-progesterone-combined sperm selection method was successfully established. After sperm were selected, the percentage of sperm with normal morphology increased (before vs. after selection, 11.2 ± 1.3% vs. 40.3 ± 6.6%, P = 0.000), the percentage of sperm with DNA fragmentation decreased (before vs. after selection, 15.4 ± 4.0% vs. 6.8 ± 3.3%, P = 0.001), and the percentage of sperm with apoptosis did not change significantly.

**Conclusions:**

Our newly-developed method is capable of successfully selecting sperm of high quality. The method will be benefit clinical ART practice as it can reduce sperm-related genetic risks.

**Electronic supplementary material:**

The online version of this article (10.1186/s12967-018-1575-7) contains supplementary material, which is available to authorized users.

## Background

Given that abnormal sperm may increase the risk of genetic defects [1–7], sperm selection is essential to ensure the health of offspring conceived via assistive reproductive technology (ART). Various sperm selection or preparation approaches have been developed for use during the ART process, including swim-up assay, density gradient centrifugation, electrophoresis, magnetic cell sorting using annexin V-conjugated microbeads, sperm morphological assessment under high magnification, hyaluronic acid bonding, microfluidics, electrophoresis, and motile sperm organellar morphology examination [8, 9]. However, these sperm processing and selection techniques bypass the natural selection process that occurs during fertilization in vivo [10].

Human sperm are subject to natural selection by a number of processes, including chemotaxis and the challenge of the long distance swim from the vagina to the fallopian ampulla in the human female reproductive system. Sperm chemotaxis is the term given to the movement of sperm in the direction of a factor (peptide or chemical) gradient [11]. It is well known that only capacitated sperm are chemotactically responsive [12, 13]. Therefore, sperm selected by chemotaxis are capacitated and may be of better quality. Progesterone secreted from human cumulus cells is a chemoattractant [14] and mediates human sperm chemotaxis [15]. Although sperm selection methods based on chemotaxis [16] and progesterone [17] have recently emerged, devices used in the various studies are very different. The essential distance from the vagina to the fallopian tube where sperm motility is selected is usually disused during the natural selection process during fertilization.

The purpose of sperm selection is to obtain better sperm quality, including sperm with normal morphology, and without DNA fragmentation and apoptosis, as these factors influence the outcomes of IVF and ICSI treatments in clinical practice. Sperm with a lower percentage of normal morphological forms is significantly related to a lower probability of ongoing pregnancies after IVF [18]. The relationship between DNA fragmentation in sperm and ART outcomes has been widely investigated, together with fertilization, embryo development, implantation, birth defects in the offspring, and early pregnancy loss [19, 20]. Furthermore, apoptotic sperm percentage has been linked with pregnancy and ART success rates [21, 22].

Therefore, the purpose of this study was to present a novel distance-progesterone-combined selection method with an original device and evaluate its efficiency for human sperm selection via the assessment of morphology, DNA fragmentation, and apoptosis. This study will provide insight into this original method for further application of clinical sperm preparation in the ART context.

## Methods

### Reagents and materials

All inorganic chemicals and agar were purchased from Sigma-Aldrich (St. Louis, MO, USA). Progesterone was acquired from ICN Biomedicals (Irvine, CA, USA). Dimethyl sulfoxide (DMSO) was obtained from Merck (Darmstadt, Germany). Human Tubal Fluid (HTF, 90126) and Serum Substitute Supplement (SSS™, 99193) were obtained from Irvine Scientific (CA, USA). Forty and 80% SpermFilter^®^ and SpermWash^®^ were obtained from Cryos International (Aarhus, Denmark). The novel designed device for sperm selection was fabricated by Qianxuan Mould Limited, Taizhou, China.

### Design and fabrication of device

**Fig. 1:**
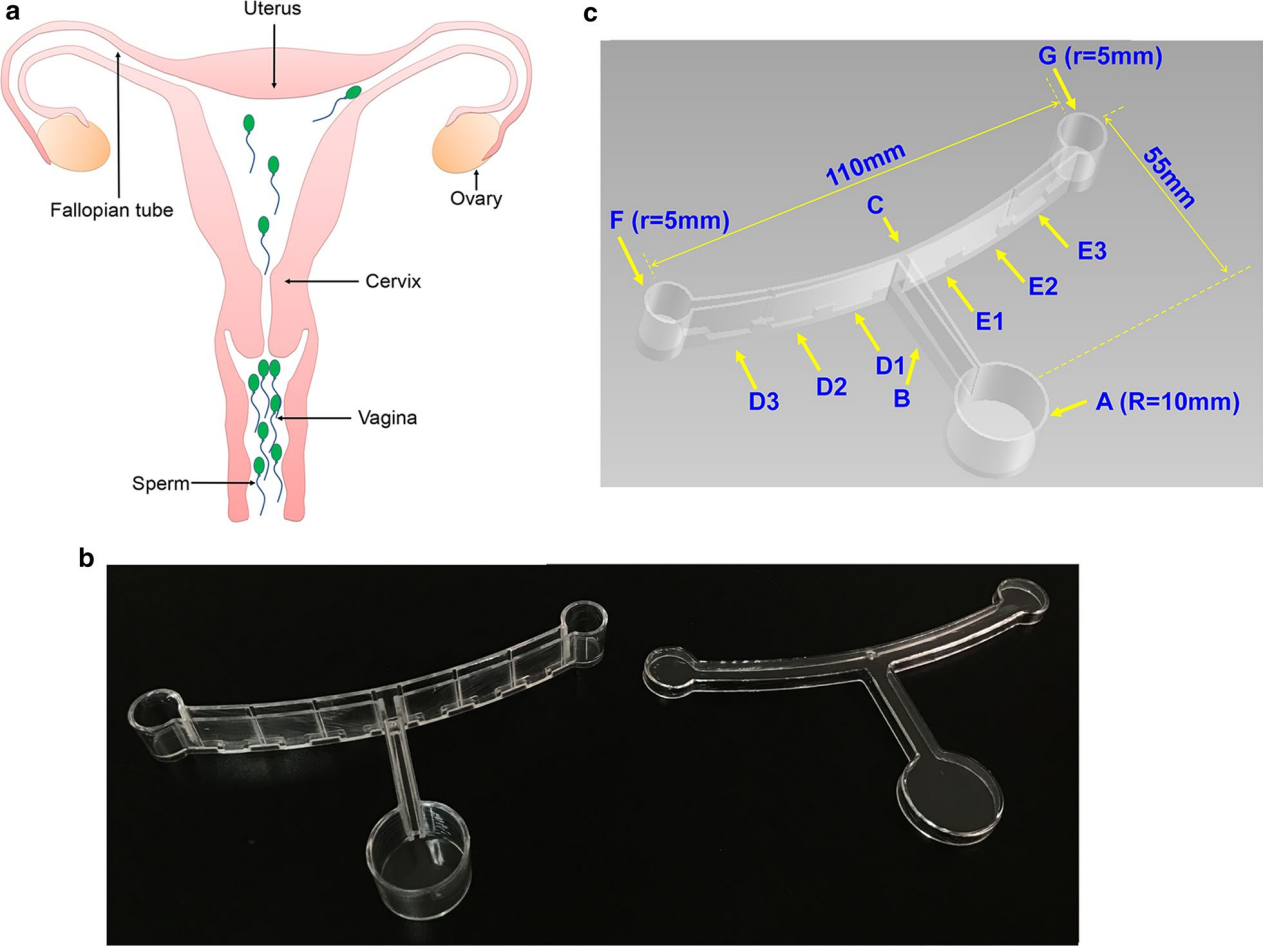
Structure of device forming chemotactic gradient. **a** Illustration of the anatomy of the female reproductive system through which sperm are naturally selected during fertilization. **b** Photograph of device; left, base of device; right, device cap. **c** Schematic diagram of the structure of the base of the device. Chamber A, sample chamber; R, the radius, 10 mm; Chamber F, control chamber; Chamber G, chemotactic chamber; r, the radius, 5 mm; Position C, the middle of the channel between Chamber F and Chamber G, similar to the uterus; Position B, the middle of the channel between Chamber A and Position C, similar to the cervix. The gap width between both walls of the channel is 2 mm, the height is 15 mm. D1, D2, and D3, grooves between Position C and Chamber F; E1, E2, and E3, the grooves between Position C and Chamber G; the dimensions of all grooves is 5 mm (length) × 2 mm (width) × 2 mm (height), the distance between neighboring grooves is 10 mm, except the interval between D1 and E1 which is 15 mm; the grooves held the agar mixed with the chemoattractant that formed the gradient

The device included a base and a matching cap (Fig. 1). The base of our device was comprised of three chambers that simulate the anatomical structures of the human female reproductive organs: Chamber A, which simulated a vagina, and a control Chamber F and a chemotactic Chamber G on either side, which simulated two ovaries. The radius of Chamber A was 10 mm, and the radiuses of both Chamber F and G were 5 mm. The channel between Chamber F and Chamber G simulated the fallopian tubes, and its center, Position C, simulated the uterus. The gap width between both walls of the channel was 2 mm and the height was 15 mm. The distance between the middle of Chamber F and Chamber G was 110 mm and the vertical distance between the center of F or G and Chamber A was 55 mm. The distances were closely approximated with the real anatomical length in the human female reproductive tract to mimic natural selection via sperm progressive motility. Position B was the middle of the channel between A and C. The grooves that held the agar mixed with the chemoattractant were set at the bottom of the channel for the forming gradient: D1, D2, and D3 at the bottom between C and F, and E1, E2, and E3 at the bottom between C and G. The dimensions of all grooves were 5 mm (length) × 2 mm (width) × 2 mm (height) and the distance between neighboring grooves was 10 mm, except the interval between D1 and E1, which was 15 mm. The device was fabricated with non-toxic food-grade polystyrene, sterilized with ethylene oxide, and sealed in a clean plastic bag until use.

### Preparation of progesterone gradient

To form the chemotactic gradient, 2% agar was used [23]. Two percent agar was prepared with an inorganic salt solution to maintain equal osmolality, containing 90 mM NaCl, 5.06 mM KCl, 25.3 mM Na_2_CO_3_, 1.17 mm KH_2_PO_4_, 1.01 mM MgSO_4_, and 50 g/ml phenol red [24]. The 2% agar was sterilized at 121 °C for 20 min before 0.03 μM progesterone in DMSO was added when needed. Then, 2% agar with the final 0.03 μM progesterone was added into Groove E1 as the chemoattractant, and only 2% agar with equal volume of DMSO into Groove D1 as the control. The devices filled with agar were kept at 4 °C inversely to avoid drying, and pre-equilibrated to 37 °C before use.

### Preparation of human sperm

Sperm preparation was performed as previously described [24, 25]. Semen specimens obtained from different healthy donors were collected by masturbation 4–7 days after abstinence and sperm parameters were examined according to WHO laboratory manual [26]. One part of the semen specimen was immediately assessed as a “Non-Percoll” sample, and the other part was separated using a discontinuous Percoll density gradient with 40 and 80% SpermFilter^®^ using centrifugation at 800*g* for 15 min. Sperm were separated from semen using Percoll separation by discontinuous density centrifugation before addition to the device. The purpose of this treatment was to remove some decapacitating factors in human semen to facilitate sperm capacitation and to ensure dead sperm without motility were detached. The supernatant was discarded, and the pellets were washed twice by the addition of 10 times the volume of Sperm Wash medium and centrifugation at 300*g* for 5 min. The final pellets were re-suspended in HTF containing 10% SSS™.

### Sperm selection

**Fig. 2:**
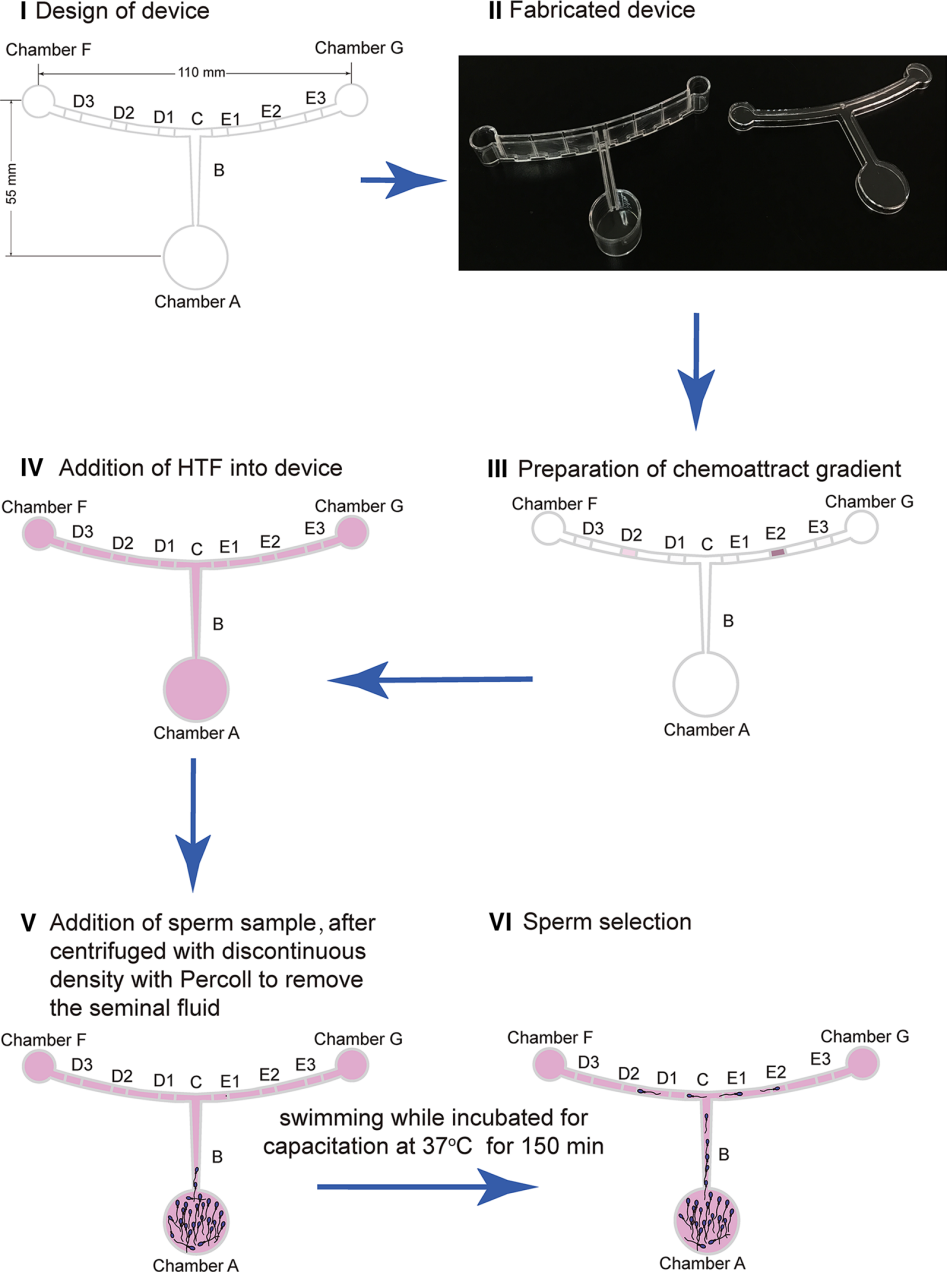
Whole workflow of sperm selection with the device. **I** Design of device; **II** device fabrication (detailed information is shown in Fig. 1). **III** Preparation of forming chemoattractant gradient; **IV** addition of HTF media into device; **V** addition of sperm sample after centrifuged with discontinuous density with Percoll to remove seminal plasma (to enable capacitation); **VI** sperm selection was performed after the sperm swam and were incubated in the device for capacitation at 37 °C for 150 min. After 150 min, sperm at Grooves D2 and E2 were pipetted for the next experiments. HTF, human tubal fluid

The prepared devices filled with the agar were immersed in 1 ml HTF media covering the base and pre-warmed with lids at 37 °C in standard humidified 5% CO_2_. According to Fick’s law, progesterone diffused into HTF and gradients formed. In total, 200 μl of prepared sperm with Percoll treatment were added into Chamber A, and allowed to swim for 150 min. The processing of swimming was also considered as selection because only sperm with progressive motility could swim a longer distance, and the chemotaxis also simultaneously acted when sperm swam near Groove E2, compared with the Groove D2 control in the absence of progesterone. At Groove D2 and Groove E2, sperm in HTF were pipetted out to carry out the next experiments. The workflow for sperm selection is illustrated in Fig. 2.

### Determination of sperm concentration

To ascertain sperm counts via the selection, sperm concentrations were determined according to the recommended procedures of the World Health Organization (WHO) laboratory manual (2010). The sperm suspension at different positions was pipetted into an Eppendorf tube with formalin fixative in NaHCO_3_ solution, and the spermatozoa were counted under a microscope using the improved Neubauer hemocytometer. To standardize the results, the percentages of the concentration at positions D and E were calculated, compared with the concentration at Chamber A at 0 min at the beginning of selection.

### Evaluation of sperm morphology

To assess the effect of sperm selection on morphology, Diff-Quik (MICROPTIC S.L. Co., Barcelona, Spain) staining was performed [26, 27]. Procedures were performed as per kit instructions. Approximately 10 μl of sperm was smeared as a thin and homogeneous layer on a clean glass slide and was air-dried at room temperature for at least 10 min. The slides were stained according to the staining procedure recommended by the manual and observed under a brightfield microscope (BH-2; Olympus, Tokyo, Japan) at 1000× magnification. Normal morphology was judged according to the WHO laboratory manual [26]. For each semen sample, at least 200 sperm per slide (or whole slides if less than 200 sperm) were counted via a double-blinded method. Then, the percentage of sperm with normal morphology was calculated.

### Sperm chromatin dispersion (SCD) test

To judge the effect of sperm selection with DNA fragmentation, sperm chromatin dispersion tests were conducted [28] with a spermatozoa SCD test (BRED Life Science Technology Inc., Shenzhen, China). The SCD test was conducted as per insert instructions. Briefly, Eppendorf tubes with low-melting point agarose were placed in a water bath at 80 °C for 20 min to fuse the agar, and kept in a water bath at 37 °C. Pre-coated slides were pre-cooled at 4 °C. Sixty microliter of the sample was added to the Eppendorf tube and mixed with the fused agar, and 30 μl mixtures were pipetted onto the pre-coated slides and rapidly covered with a 22 × 22 mm coverslip. The slides were placed on a cold plate in the refrigerator (4 °C) for 5 min to allow the agar to produce a microgel with the sperm cells embedded within. The coverslips were gently removed, and the slides were immediately immersed horizontally in an acid solution, previously prepared as a mixing reaction solution A, and incubated for 7 min. The slides were horizontally immersed in reaction solution B for 25 min. After washing for 5 min in a tray with abundant distilled water, the slides were dehydrated in increasing concentrations of ethanol (70–90–100%) for 2 min each, then air-dried. Slides were covered horizontally with a mix of Wright’s staining solution provided in the kit with continuous airflow for 15 min, and the slides were briefly washed in distilled water and allowed to dry. Then, a minimum of 200 sperm or whole slides (if less than 200 sperm) per sample were observed under the 400× objective of the microscope (BH-2; Olympus). Whether sperm contained fragmented DNA depended on the SCD patterns of sperm. According to the instructions in the kit, sperm with halo widths equal to or smaller than one-third of the minor diameter of the core contained fragmented DNA. Finally, the percentage of sperm with fragmented DNA was calculated.

### Assessment of sperm apoptosis status

To evaluate the effect of sperm selection on apoptosis status, an annexin V-FITC/PI method was employed [29] with an annexin V-FITC/PI Detection Kit (Yeasen Science Technology Inc., Shanghai, China). The sperm were washed twice with PBS by centrifugation at 300*g* for 5 min, and the pellets were suspended in 1× binding buffer, then mixed with annexin-V-FITC (annexin V-FITC/PI), and reacted for 10 min. The binding buffer was added before the sample was placed on the slides. The slides were covered with cover glasses and observed under the fluorescent microscopy (Nikon Eclipse 80i, Tokyo, Japan) with a 450–500 nm excitation wavelengths and 515–565 nm emission wavelengths. Sperm were classified as normal with negative annexin and PI in red, apoptotic with positive annexin-V in green, and negative PI. Two-hundred spermatozoa or whole slides (if less than 200 sperm) were randomly assessed per sample and identified as normal or apoptotic. The percentages were calculated.

### Statistical analysis

Results were expressed as mean ± SEM. Data were analyzed using PASW Statistics 18 (SPSS Inc., Chicago, IL). Normal distribution of data was determined by the Kolmogorov–Smirnov test or Shapiro–Wilk test. When study variables were normally distributed, data were analyzed using a paired *t* test or an independent samples *t* test, or data were analyzed using the Mann–Whitney U test. P values < 0.05 were considered statistically significant.

## Results

### Proportion of sperm after selection with the distance-progesterone-combined method

**Fig. 3:**
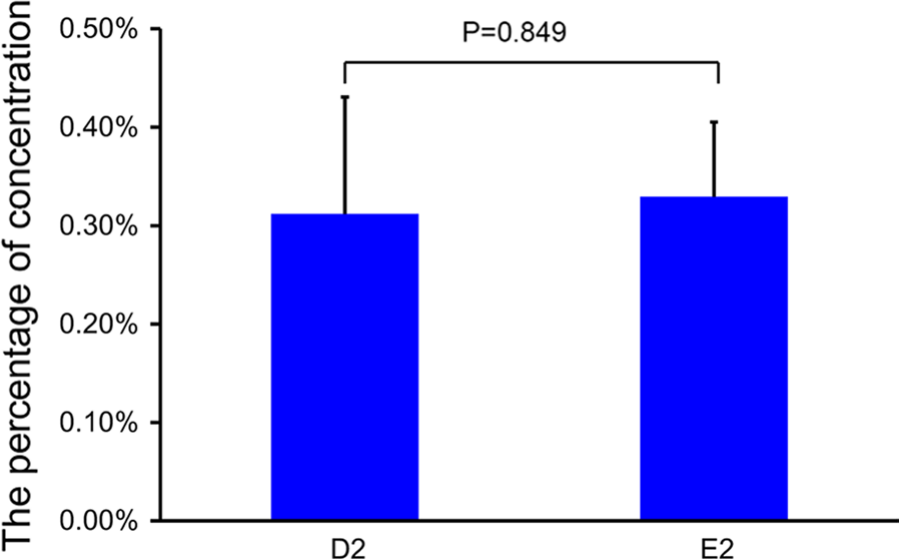
The percentage of sperm concentration at different positions. D2, Position Groove D2; E2, Position Groove E2. When selected, the fixed 200 μl of sperm suspension was put in Chamber A, the original concentrations at Chamber A at 0 min represented 100%. Data are expressed as mean ± SEM (n = 8). The results suggest that sperm are able to swim to positions D2 and E2 from Chamber A in 150 min and the majority of sperm were screened. There was no statistical difference between Positions D2 and E2 (paired t test, P = 0.849)

The sperm concentrations in positions D2 and E2 relative to the original position, Chamber A, were measured to determine how many sperm could be acquired after selection. Sperm were able to swim to positions D2 and E2 at 150 min (Fig. 3). The sperm concentration at position D2 did not differ from that at position E2 (P = 0.849).

### Effect of selection with the distance-progesterone-combined method on sperm morphology

**Fig. 4:**
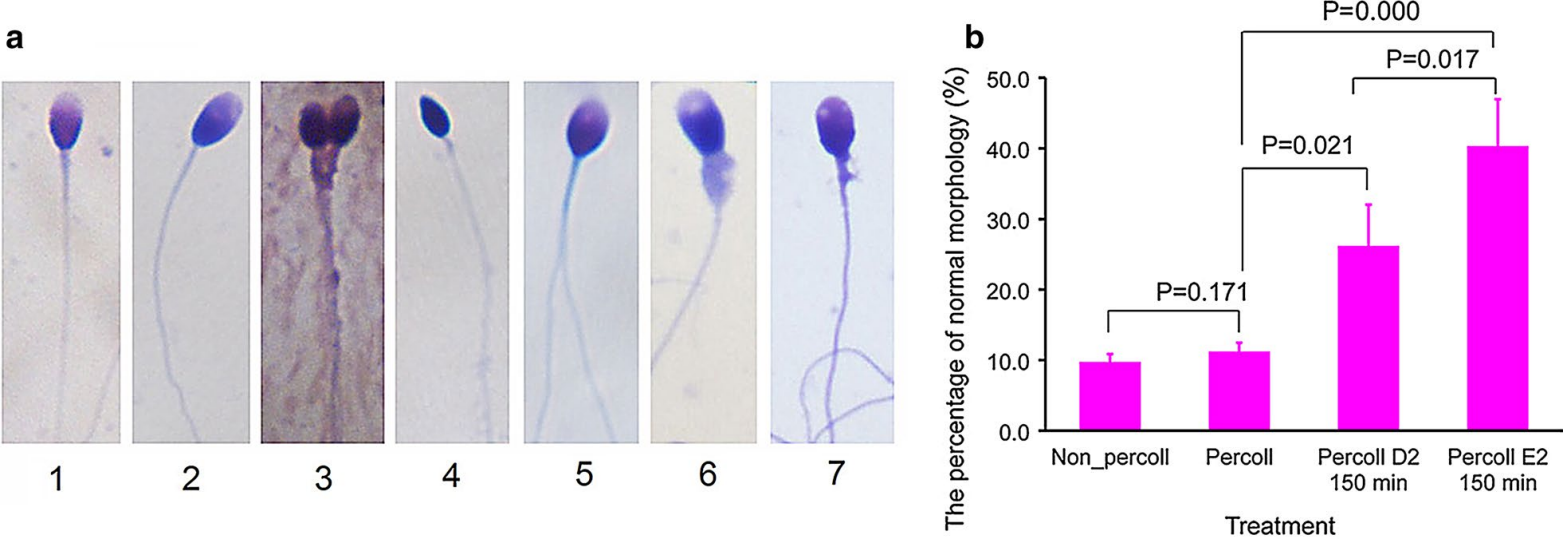
Effect of selection combined-distance-progesterone on sperm morphology. **a** Part of sperm morphology: 1, normal; 2–7, abnormal. The bar represents 5 μm. **b** The percentage of sperm with normal morphology after different treatments. Data are expressed as mean ± SEM. There was a significant difference between D2 and E2 (paired t test, P = 0.017, n = 19), and no significant difference between Non_Percoll and Percoll (P = 0.171, n = 28). Non_Percoll indicates the sperm were not processed by Percoll before selection; Percoll, the sperm were processed by Percoll before selection; D2 and E2, position of device. Min, minutes; P, P value

Sperm morphology, as assessed by Diff-Quik staining, is displayed in Fig. 4. Before sperm selection, the percentage of sperm with normal morphology did not differ according to whether or not Percoll treatment was applied (P = 0.171). However, the percentage of sperm with normal morphology at position E2 was significantly higher (independent samples *t* test, P = 0.000) after selection compared to before selection. Similarly, the percentage of sperm with normal morphology at position D2 was significantly higher (independent samples *t* test, P = 0.021) after selection compared to before selection. Furthermore, the difference between E2 and D2 was also statistically significant (P = 0.017).

### The effect of selection with the distance-progesterone-combined method on sperm containing DNA fragmentation

**Fig. 5:**
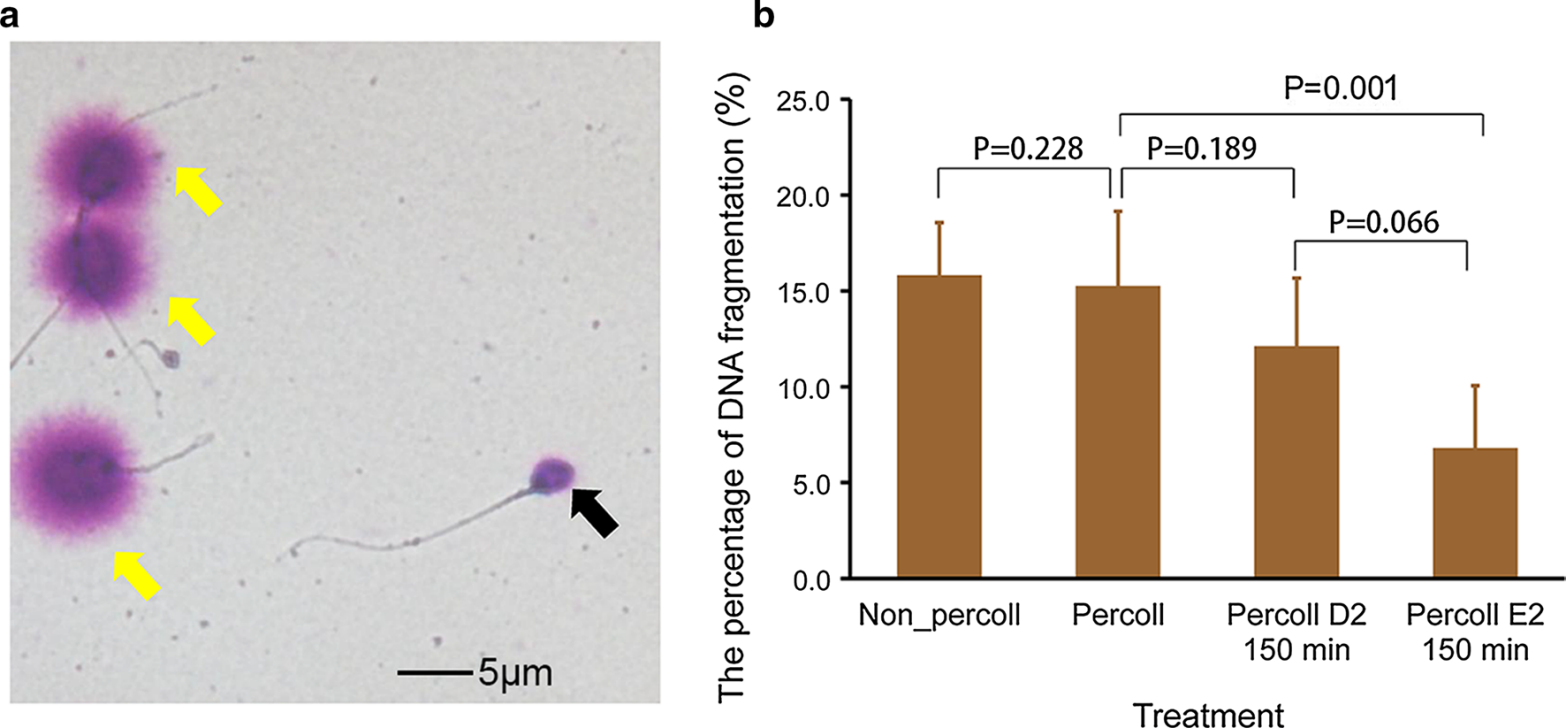
Effect of selection combined-distance-progesterone on sperm containing DNA fragmentation. **a** Sperm with integrated DNA (yellow arrows) and DNA fragmentation (black arrow). The bar represents 5 μm. **b** The percentage of sperm with DNA fragmentation after different treatments. Data are expressed as mean ± SEM. There was a significant difference between the percentage of sperm with DNA fragmentation at position E2 and with Percoll treatment before selection (Mann–Whitney U test, P = 0.001), and no difference between sperm treated with Percoll and those that were not (P = 0.228, n = 28). However, there was no statistical difference between position D2 and E2 after selection (P = 0.066, n = 23). Non-Percoll indicates that the sperm were not processed with Percoll before selection; Percoll, indicates that the sperm were processed by Percoll before selection; D2 and E2, positions on the device. Min, minutes; P, P value

Sperm with DNA fragments or integrity are shown in Fig. 5. Before selection, the percentage of sperm containing DNA fragmentation did not differ according to whether or not Percoll treatment was applied (P = 0.228). However, the percentage of sperm containing DNA fragments at position E2 after selection was lower (P = 0.001) in sperm treated with Percoll treatment before selection. Sperm containing DNA fragments at position D2 did not differ (P = 0.189) from sperm treated with Percoll before selection. Meanwhile, the difference between D2 and E2 was not statistically significant (P = 0.066).

### Effect of the distance-progesterone-combined method on sperm apoptosis

**Fig. 6:**
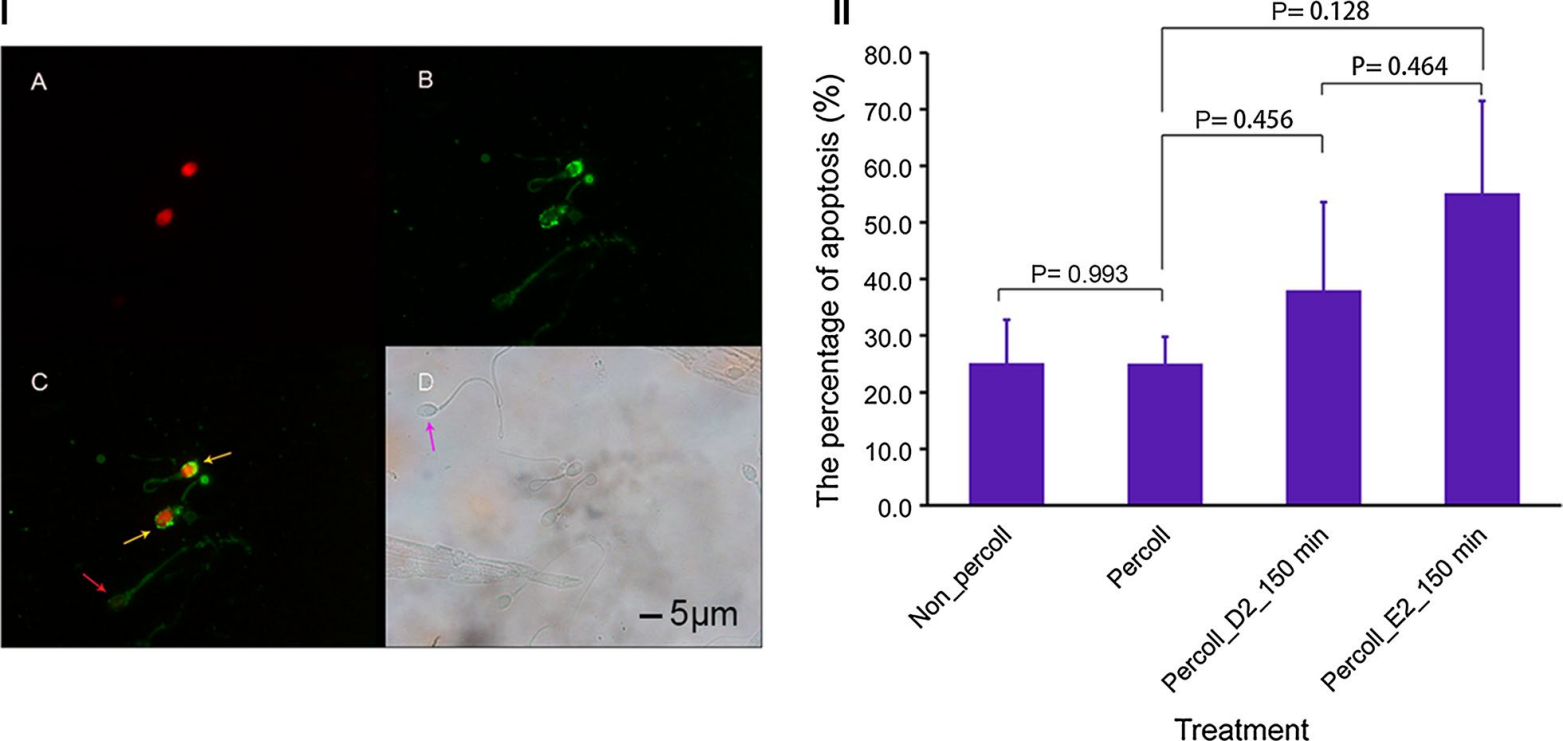
Effect of selection combined-distance-progesterone on sperm apoptosis. **A** Sperm nucleus (red); **B** annexin V on the surface of sperm (apoptotic, green); **C** late (yellow arrow) and early (red arrow) apoptotic sperm; **D** nonapoptotic sperm (pink arrow). **I** The bar represents 5 μm. **II** The percentage of apoptotic sperm after different treatments. Data are expressed as mean ± SEM (n = 6). There was no significant difference between sperm treated with Percoll and sperm not treated with Percoll (independent samples t test, P = 0.993), or between positions D2 and E2 (P = 0.464). Non-Percoll indicates the sperm were not processed by Percoll before selection; Percoll indicates sperm were processed by Percoll before selection; D2 and E2, position of device. Min, minutes; P, P value

Sperm apoptosis status and results are shown in Fig. 6. The percentage of apoptosis in sperm treated with Percoll was not statistically different (P = 0.993) from sperm not treated with Percoll. In sperm treated with Percoll, the percentage of sperm with apoptosis at position E2 (P = 0.128) or at position D2 (P = 0.456) did not differ significantly before and after selection. Furthermore, there was no significant difference between the percentage of sperm with apoptosis at position E2 and at position D2.

## Discussion

In this study, we investigated the relationship between swim distance, sperm motility, and chemotaxis by progesterone in the human female reproductive tract during natural selection. As the designed device (as shown in Fig. 2) imitated the real distance between the vagina, uterus, fallopian tubes, and ovaries in the human female reproductive system, only sperm with progressive motility could swim the long distance and sperm with poor motility could be removed. The results showed that less than 0.50% sperm could swim to the Groove D2 position or Groove E2 position in 150 min along the channel of the device when samples were added at Chamber A. The results suggest that this sperm selection method is capable of removing an overwhelming majority of sperm, leaving only a small portion of sperm that move forward. The results are consistent with the phenomenon that occurs during natural fertilization, in that only a few capacitation sperm reach the oocyte-containing tube during natural fertilization [30]. Additionally, using this method with an even longer distance may remove most sperm with inferior motility, and is therefore more effective than the swim-up method, because the sperm prepared with the swim-up method usually swim a shorter distance with a small volume in a test-tube. The reason may be that the device has a length equivalent to the human female reproductive tract allowing the sperm to migrate for further examination of physiological status through the selection pressure during migration [31]. Hence, mimicking the real distance during natural fertilization was helpful and beneficial for human sperm selection. Given that the size of the microfluid chip is much smaller than the real human female anatomic structure, the chip method [16] for sperm selection may not be optimal. On the other hand, sperm chemotaxis using progesterone during selection was also deliberated in this method. Only the sperm that were already very close to Groove E2 were selected by chemotaxis. There were certain conditions that attracted sperm to Groove E2: (1) the distance between Groove E2 and E1, which contained progesterone, was very close (only a couple of millimeters) [11], (2) sperm there were capacitated because they swam for 150 min under capacitation culture conditions (also incubated in HTF media at 37 °C in a standard humidified 5% CO_2_), and (3) the timing of human sperm capacitation was programmed with continuous replacement [32]. Moreover, the number of sperm that swam to Groove D2 was not statistically different to the number that swam to Groove E2 (Fig. 1), although the chemoattractant progesterone was added into Groove E1. However, progesterone action could not be ruled out, although it seemed that progesterone failed to attract more sperm during the sperm selection period. The main reason for this may be that the proportion of capacitated sperm with a chemotactic response was very small, which could not be differentiated from other causes of sperm accumulation on the basis of sperm counts [11]. Thus, the results show that most sperm were screened out and only a small portion of sperm were selected with our established method, and the purpose of sperm selection was achieved.

Several important experimental factors on sperm selection were of concern in the present study. First, progesterone was used as a chemoattractant to closely mimic the natural physiological processes because it is commonly secreted from cumulus cells when sperm meet the egg [14], although a variety of different chemoattractants have been reported. Second, only Groove D2 and E2, and not all grooves at the bottom of the designed device were used because the combined conditions of different concentrations of progesterone and different grooves had been validated and optimized (see Additional file 1). Third, sperm were subjected to Percoll separation by discontinuous density centrifugation before addition to the device. The purpose of this treatment was mainly to remove some decapacitating factors in human semen to facilitate sperm capacitation and ensure dead sperm without any motility were detached. Finally, the time for sperm selection depended on the time it took for sperm to swim and reach Groove E2 and the suitable 1–4 h time for human sperm capacitation that is vital for sperm chemotaxis. This is because the capacitated and chemotactic responsive state is transient and short [11, 30, 33]. Meanwhile, the time should be as short as possible when preparing sperm for ICSI or IVF procedures. Overall, the present study has successfully established a new sperm selection method.

Our evaluation of the performance of our distance-progesterone-combined selection method showed that this method enhances the proportion of sperm with normal morphology and lowers the proportion of sperm with DNA fragmentation. The results showed more sperm with normal morphology and more sperm with DNA integrity were acquired on the progesterone treatment side of the device. Furthermore, morphology was involved in chemotaxis by progesterone. As shown in Fig. 4, the effect of the swimming long distance on morphology selection shows significance (P = 0.021). Similarly, the effect of chemotaxis (P = 0.017), and the combined effect of swimming long distance and chemotaxis also showed high significance (P = 0.000). However, DNA fragmentation selection is different from morphology selection. The combined conditions of swimming long distance and chemotaxis is required. The results showed that only the combined effect of swimming long distance and chemotaxis showed a significant difference (P = 0.001) (Fig. 5), not swimming long distance alone or chemotaxis alone. Importantly, increasing the proportion of sperm with DNA integrity is consistent with the results of progesterone selection in a previous report [17]. The reason why the sperm quality after selection is improved is that spermatozoa with abnormal morphology and/or fragmented DNA do not function properly so they probably do not undergo capacitation and are not chemotactically responsive. Furthermore, results showed that this selection method does not change the proportion of sperm with apoptosis (P = 0.128). This implies that this method has no adverse effects on sperm. Additionally, apoptosis is not related to chemotaxis by progesterone, because the difference in sperm apoptosis with and without progesterone treatment was not different (P = 0.464).

Overall, the method used in the present study can improve sperm quality and benefit sperm preparation. Moreover, the results suggest it is possible that there is a relationship between sperm morphology, DNA fragmentation, and sperm motility and/or chemotaxis, but there is no direct evidence. Therefore, the questions raised from this study need further investigation in future research.

To the best of our knowledge, this is the first time a distance-progesterone-combined selection method for human sperm has been reported. The advantage of this method is that it is capable of scrutinizing biological sperm functions that are very important for fertilization, not merely the physical and chemical characteristic(s) of sperm. Moreover, the results and conclusion in this study are more applicable to human ART compared to studies evaluating mouse sperm [16]. Since the design of this method depends on the normal physiological conditions during fertilization, this method is more suitable for scenarios with normospermic semen: in oligozoospermia specimens, very few sperm (approximately 0.3% as shown in Fig. 3) after selection is not sufficient for the next stage of the ART procedure; for asthenospermia specimens, sperm with very poor motility may fail to reach the end of the selection position in the device. Without sperm selection in ART treatment, the incidence of spontaneous abortion [34] and genetic risk [35] may increase. Moreover, normospermic semen is the most common case of not only unexplained male infertility, but also female infertility. Therefore, this selection method that provides an effective solution to this will have wide application in clinical practice. Undoubtedly, only the distance and progesterone chemotaxis were mimicked and other aspects of the complete microenvironment of the female reproductive tract were not considered in this study.

## Conclusions

This novel sperm selection method based on distance-progesterone successfully improved human sperm quality. The cost of this method is low because expensive equipment is not needed, and the procedure is easy to perform. Therefore, this sperm selection method is promising for application in clinical ART treatment to reduce sperm-related genetic risks.

## Additional file

**Additional file 1.** Validation and optimization of experimental conditions for the distance-progesterone-combined sperm selection method. The additional file shows the validation and optimization for the combined conditions of different progesterone concentrations, different positions, and experimental times for the distance-progesterone-combined sperm selection method.

## Abbreviations

ART: assistive reproductive technology
Annexin-V-FITC/PI: annexin V-fluorescein isothiocyanate/propidium iodide
DMSO: dimethyl sulfoxide
HTF: human tubal fluid
IVF: in-vitro fertilization
ICSI: intracytoplasmic sperm injection
SCD: sperm chromatin dispersion
Min: minutes
SEM: standard error of mean
WHO: World Health Organization
