# Supplementary material for: Novel distance-progesterone-combined selection approach improves human sperm quality
Source: J Transl Med. 2018 Jul 20;16:203. doi: 10.1186/s12967-018-1575-7 (PMC6053761; doi:10.1186/s12967-018-1575-7)
Supplement: Supplementary file 1 — Additional file 1. Validation and optimization of experimental conditions for the distance-progesterone-combined sperm selection method. The additional file shows the validation and optimization for the combined conditions of different progesterone concentrations, different positions, and experimental times for the distance-progesterone-combined sperm selection method. [file 12967_2018_1575_MOESM1_ESM.docx]

**Validation and optimization of experimental conditions for the distance-progesterone-combined sperm selection method**

**Method**

**Preparation of chemoattractant gradient**

The protocol was the same as described in “Preparation of chemoattractant gradient” in the main text, apart from the positions and concentrations of progesterone in the device set, as Table S1 describes.

**Preparation of human sperm**

The protocol was the same as described in “Preparation of human sperm” in the main text.

**Calculation of concentration percentages**

The prepared devices filled with the agar were immersed in 1 ml HTF media covering the base, and pre-warmed with lids at 37^o^C in standard humidified 5% CO_2_. In total, 200 μl of prepared sperm with Percoll treatment was placed into Chamber A, and allowed to swim for different lengths of time (30 min, 60 min, 90 min, 120 min, 150 min, 180 min, 210 min, 240 min, 270 min, or 300 min). At different positions (Position B, C, D2, E2, F, or G), sperm in HTF were pipetted out to determine the concentration according to the “Determination of sperm concentration” in the main text. To standardize the results, the percentages of the concentrations at different positions at corresponding times were calculated and compared with the concentration at Chamber A at 0 min.

**Results**

**Concentration percentages at Position B under different experimental conditions**

To explore the situation, sperm swam in the device under conditions P1, P2, and P3, and the concentrations at Position B were determined at 30, 60, and 90 min, respectively. The percentages of concentrations were calculated and compared with the concentration at Chamber A at 0 min. The results (Figure S1) showed sperm could reach Position B after swimming for only 30 min. Furthermore, the amount of sperm gradually decreased over time (30 min to 90 min).

**Concentration percentages at Position C under different experimental conditions**

Similar to Position B, the concentrations at Position C were determined at 30, 60, and 90 min. The percentages of concentrations were calculated and compared with the concentration at Chamber A at 0 min. The results (Figure S2) showed sperm could reach Position C after 30 min. Furthermore, the amount of sperm gradually increased over time (between the 30 min and 90 min time points).

**Concentration percentages at Position D2 and E2 under different experimental conditions**

The results (Figure S3) showed sperm also could be detected at Position D2 and E2 from 90 min to 210 min. However, it was evident that the amount of sperm at each time point was much lower than that at Position B and C. Also, the increasing or decreasing trend at Position D2 and E2 was not significant with time extension, unlike Position B or C.

**Concentration percentages at Position G and F under different experimental conditions**

Like the results of Position D2 and E2, our results (Figure S4) revealed sperm could also be detected at Position G and F after swimming for 150 min to 300 min. Furthermore, the amount of sperm at each time point was similar to the results at Position D2 and E2, which were much lower than that at Position B and C. Significant increasing or decreasing trends, such as in Position B and C, were difficult to observe with time extension.

**Discussion**

All test conditions for sperm selection in this study were validated by experiments. To validate the situation, sperm swam under the different experimental conditions, and the amounts of sperm at different time points at different positions were observed. The amounts of sperm at Position B gradually decreased between the 30 min time point and 90 min time point (Figure S1). This suggests sperm moved on from Position B with the extension of time. At the corresponding times, the amount of sperm at Position C gradually increased (Figure S2). This indicates that sperm swam to Position C from B. At Position D2 and E2, more time points and longer time points were investigated because the distance from Chamber A was farther than that of Position B and C. The results show that the amount of sperm at each time point was much lower (Figure S3) than those at Positive B and C. This indicated the distance factor screened sperm out and therefore, distance is important for sperm selection. Five longer time points at Positions F and G were examined because they were the farthest distances (Figure S4). The amount of sperm at each time point was much lower, similar to Position D2 and E2. It may suggest the longer time at Position F and G could not increase the sperm amount.

Therefore, the following rules were observed: 1) under P1, P2, or P3 conditions, sperm could swim to Position F and G, at the farthest end of the device, 2) sperm counts decreased with longer distances from Chamber A, 3) under the P1 condition, the amounts of sperm in the majority of groups at Positions D2, E2, F, or G were more than those under the P2 or P3 condition, and 4) with longer times, sperm amounts at Position F and G were not greater than sperm amounts at Position D2 and E2.

Based on the rules observed in the validated experiments, sperm were placed in Chamber A to swim to Position E2 for 150 min under the P1 condition. A time of 150 min was chosen because: 1) 150 min enables sperm to be capacitated sufficiently and 2) 150 min was suitable for application in the clinical context.

In conclusion, the experimental conditions for the distance-progesterone-combined sperm selection method in this study have been validated and optimized. As such, these conditions will be applied: a chemoattractant gradient with 0.32 µM progesterone set in Groove E1 in the device and sperm placed in Chamber A to swim to Position E2 for 150 min.

**Figure Legends**

Figure S1. Concentration percentages at Position B under different experimental conditions

The figure shows the concentration percentage of sperm that swam to Position B (B) at 30, 60, and 90 minutes (Min) after Percoll (Perc) treatment, compared with the concentration at Chamber A at 0 min. P1, P2, and P3 indicates the progesterone conditions listed in Table S1. Experiments were repeated 3-10 times.


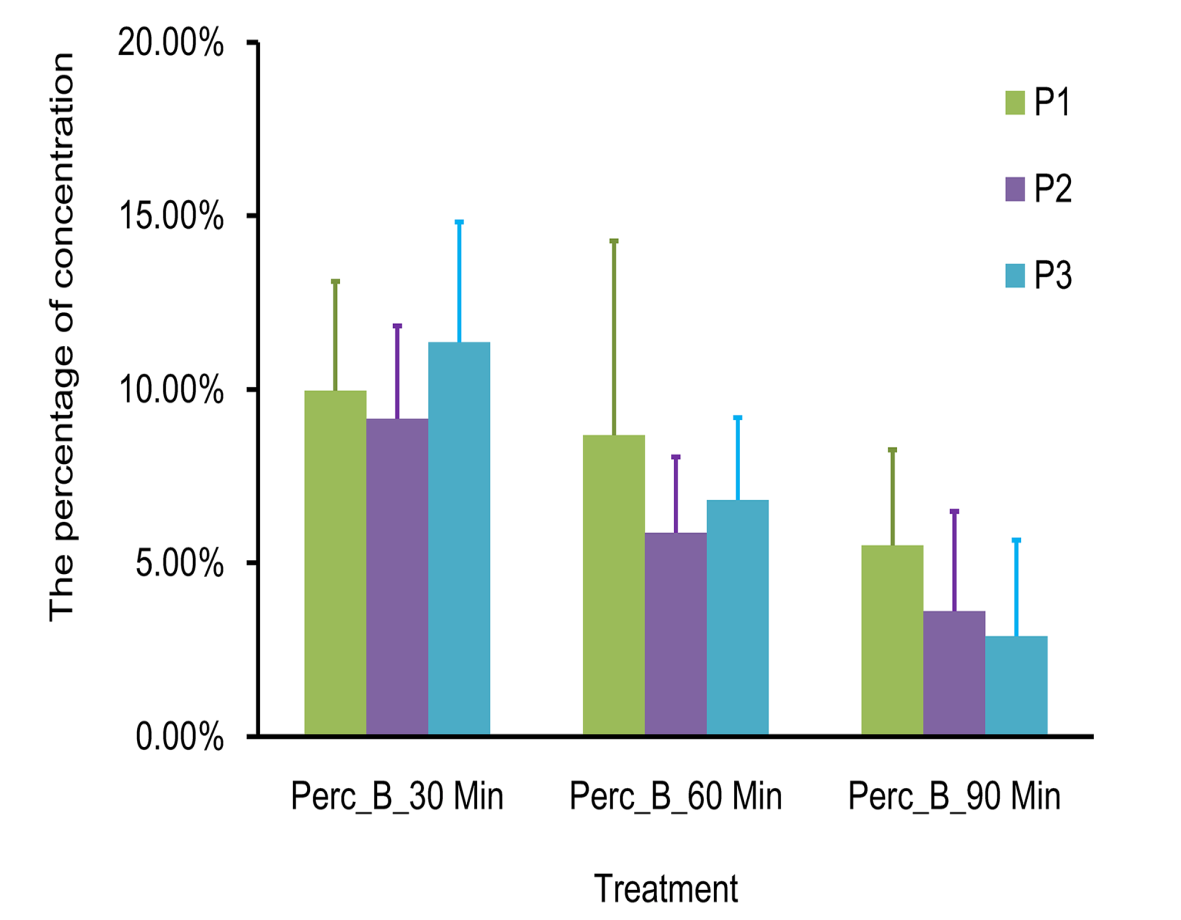


Figure S2. Concentration percentages at Position C under different experimental conditions

The figure shows the concentration percentage of sperm that swam to Position C (C) at 30, 60, and 90 minutes (Min) after Percoll (Perc) treatment, compared with the concentration at Chamber A at 0 min. P1, P2, and P3, including the progesterone conditions listed in Table S1. Experiments were repeated 3-10 times.


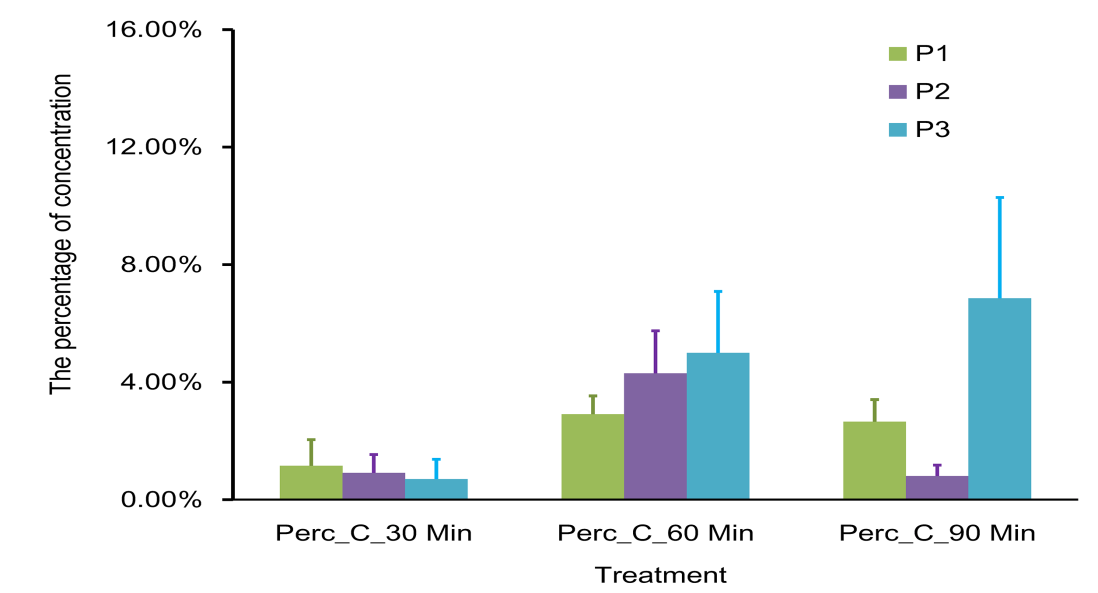


Figure S3. Concentration percentages at Position D2 and E2 under different experimental conditions

This figure shows the concentration percentage of sperm that swam to Position D2 (D2) and E2 (E2) at 90, 120, 150, and 210 minutes (Min) after Percoll (Perc) treatment, compared with the concentration at Chamber A at 0 min. P1, P2, and P3 indicated the progesterone conditions listed in Table S1. Experiments were repeated 2-9 times.


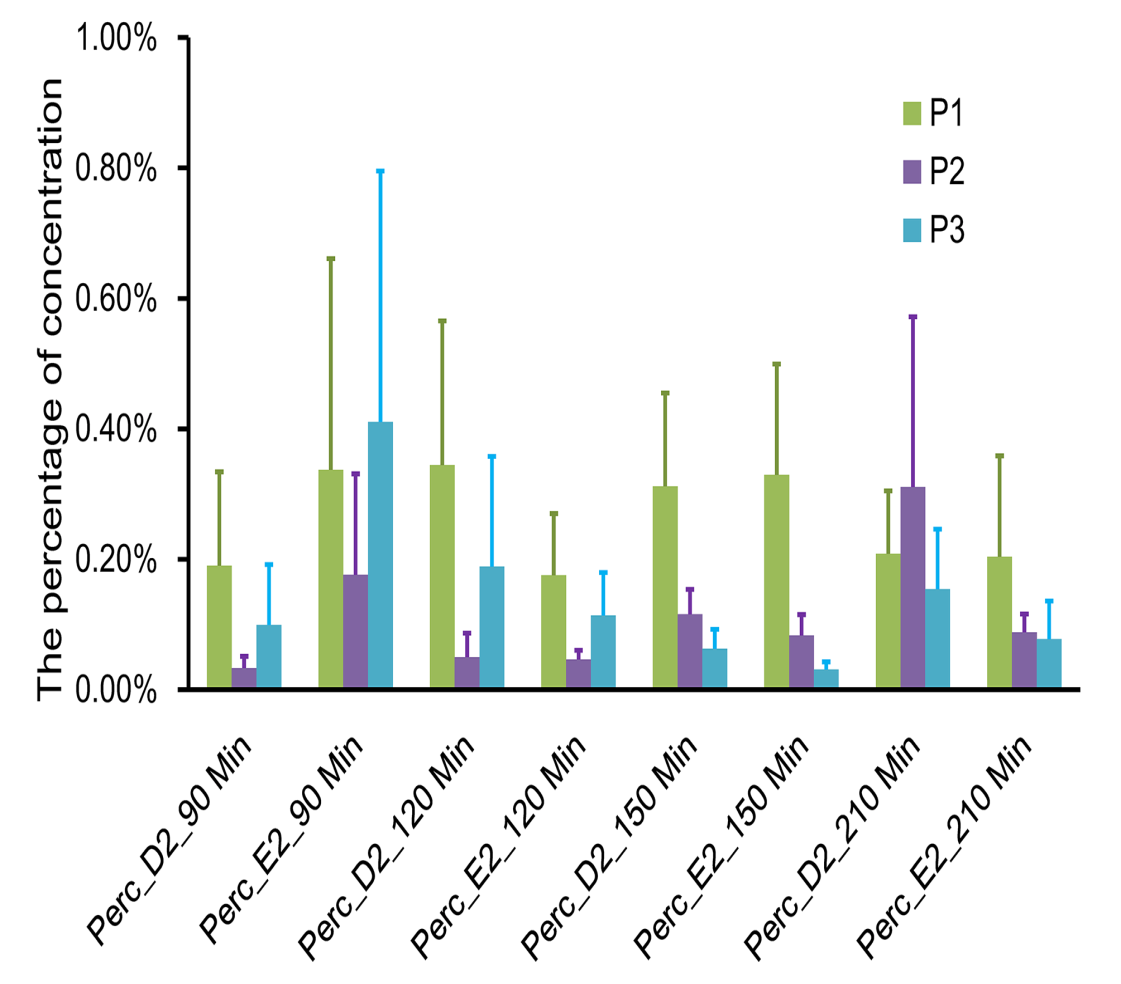


Figure S4. Concentration percentages at Position F and G under different experimental conditions

This figure shows the concentration percentage of sperm that swam to Position F (F) and G (G) at 150, 180, 210, 210, 240, and 300 minutes (Min) after Percoll (Perc) treatment, compared with the concentration at Chamber A at 0 min. P1, P2, and P3 as indicated in the progesterone conditions listed in Table S1. Experiments were repeated 2-7 times.


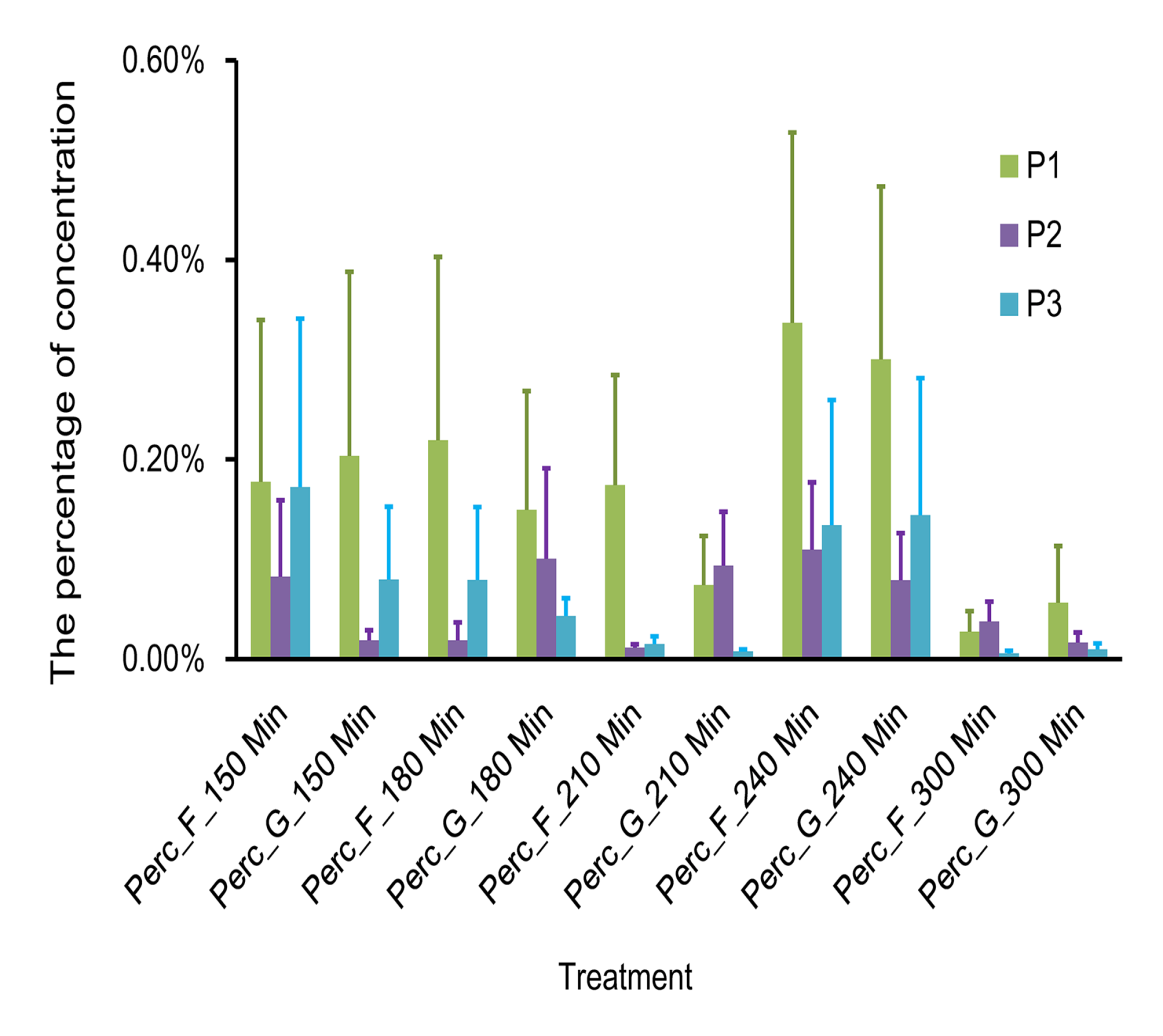


**Table S1. Positions and concentrations of progesterone in device**

|  | Control side | | |  | Chemotactic side | | |
| --- | --- | --- | --- | --- | --- | --- | --- |
|  | Groove D3 | Groove D2 | Groove D1 |  | Groove E1 | Groove E2 | Groove E3 |
| Condition 1 (P1) | - | - | DMSO |  | 0.32 µM  progesterone | - | - |
| Condition 2  (P2) | - | DMSO | - |  | - | 0.32 µM progesterone | - |
| Condition 3 (P3) | DMSO | DMSO | DMSO |  | 0.32 µM  progesterone | 0.63 µM  progesterone | 1.25 µM  progesterone |

DMSO, dimethyl sulfoxide
